# Kin term mimicry hypothesis

**DOI:** 10.1007/s12064-023-00393-1

**Published:** 2023-06-05

**Authors:** Bogusław Pawłowski, Anna Chmielińska

**Affiliations:** Department of Human Biology, University of Wrocław, ul. Przybyszewskiego 63, 51-148 Wrocław, Poland

**Keywords:** Mimicry, Kinship, Linguistic evolution, Cooperative behavior, Reciprocity

## Abstract

Adaptive mimicry in animals is a well-known phenomenon. Here, we propose that a similarly adaptive strategy in humans is using kin terms for people who are not closely genetically related. Irrespective of the initiator attributing a kin term to a non-kin, we call this kin term mimicry (KTM). The emergence of human sociality and language allowed not only easy kin recognition, but also led to strong positive emotions related to such kin names as “mother,” “father,” “brother,” “sister,” “aunt” or “uncle.” Although the phenomenon of using kin terms of genetically unrelated people is well known in the social sciences, here we discuss it in the light of evolution. We notice this is an evolutionary adaptive cooperation strategy, which allows us to predict in which ecological or social circumstances it will be more prevalent. We postulate specific testable factors that affect the prevalence of kin mimicry. We also discuss who is more likely to be an initiator of calling non-kin a fictive kin, and who benefits from such behavior. The KTM hypothesis postulates that an individual or social group initiating or bestowing kin terms usually receives more benefits (economic and/or psychological support) from such mimicry.

Mimicry occurs when one species changes in time to resemble another species or object, in order to confuse their potential prey or predators. It is a well-known phenomenon in many organisms (Edmunds and Golding 1999). Even though mimicry is often imprecise, it exemplifies the mechanism of natural selection (Edmunds and Golding 1999) and is a quite frequently observed evolutionary strategy in the animal kingdom (Kelley et al. 2008; Moore and Hassall 2016). It allows either to protect an organism against potential predation (Maran 2017) or in the case of predators, to pretend to be another non-threatening organism. Examples of mimicry can also be found within plants (Schaefer and Ruxton 2009), or even in artificial neural networks, such as in an experiment where three populations (two senders and a receiver) of artificial neural networks were allowed to co-evolve. After 50,000 generations the mimic changed in time, resembling the model, to deceive the receiver (Holmgren and Enquist 1999). Mimicry would thus seem to be quite a common phenomenon, usually related to an organism’s adaptation.

Interestingly, mimicry does not always serve competition as traditionally understood in the case of prey–predator systems (Batesian mimicry). It can be also used to enhance cooperation [Müllerian mimicry (Edmunds and Golding 1999)] or in mating. It is the case, for instance, in the context of mating that when approaching a female, a male pretends to be a female (Straaten et al. 2008).

Such defined mimicry can be therefore understood as a type of social interaction where a “sender” sends some mimicry to communicate, and the “receiver” receives it.

The within-species mimicry also occurs in humans. For instance, culture allows people (e.g., soldiers or warriors) to mirror a specific environment in order to hide themselves from an enemy (Newark 2007). Military organizations or religious cults may train their recruits to look alike, so they bond easier to each other (Qirko 2004). Kinship terminology might be then used to reinforce the attachment between recruits (brothers in arms, buddies of the battlefield, brides of Christ) and to establish dominance or manipulate an entity to sacrifice a lot (Qirko 2009). People might, for instance, use clothes and foreign language to pretend they are from another group or society, and this might be done in order to spy on an enemy. To increase mating success, some people also pretend to have more resources, or undergo cosmetic surgery or use strong make-up to appear younger or more fertile (Lirola and Chovanec 2012). Mimicry is common to the point that it happens “automatically” and with the phenomenon being noticed neither by sender nor receiver. Not all mimicry is apparent to the human eye (Grim 2013), and in the case of humans it does not need to involve a visual change. A known social phenomenon is for example unconscious usage of similar vocabulary, or making a similar pose to a mimicked person (Lakin et al. 2008; Monin 2003). Besides the above mentioned potential in-group mimicry in mating or war, there is, however, no other distinguished and well described mimicry in humans. Here we propose a new species-specific adaptive form of mimicry in humans that we call kin term mimicry (KTM).

By kin term mimicry we refer to the situation when a person, who is not a close relative, is called by a close kin name (e.g., mother, father, brother, sister, uncle). Acknowledging the existence of six different kin terminology systems (Cronk et al. 2019), we describe our view using the Eskimo^1^ system as a template. Kin term mimicry may be considered to exist in the case of any use of a kin name which is not a real name or a true description of a real kinship, and which pretends to denote stronger genetic affinity compared to an existing one. For example, a real aunt has a 25% genetic relatedness to a nephew. But when called a godmother, the affinity between the aunt and the nephew evokes connotations of a relationship with a 50% genetic relatedness. We suggest the phenomenon of the KTM is sufficient to develop a relationship which can positively influence an individual’s fitness. This means that KTM may have an impact on an economic and/or social status of an individual that usually initiate calling a non-kin (e.g., a friend or a priest) with a kin term (e.g., aunt or father, respectively), or calling more distant kin (e.g., a distant cousin) with a closer kin name (an aunt or godmother). We believe that bestowing a certain kin name leads to the possibility of fictive kin being treated according to Hamilton's rule (Hamilton 1964), and therefore we postulate that KTM in humans is a mechanism that promotes cooperation and can be an evolutionarily adaptive phenomenon.

In the majority of societies in the world, people sometimes use kin naming towards people with whom they do not have genetic relatedness. As children learn to speak, the first vocabulary they use are those that denote their closest relatives who give them care, and with whom they can bond. Salmon (1998) noticed that if such kin denoting vocabulary is used towards unrelated entities, it exploits the usual emotional bond and obligations that normally exists between real kins. The most common KTM is related to the custom of having god-parents, with conferring the name “aunt” or “uncle” upon the close friends of a child’s parents, naming a priest a “father”, or a nun a “sister.” In the case of the Christian church, priests are trained to use kinship terminology in relation to the institution (Qirko 2004). Furthermore in cohesive communities, such as in social communes, people call themselves “sister” and “brother” (Dunbar 2010; Abou-Abdallah et al. 2016). It is worth noticing that kin term mimicry is basically restricted only to the kin with genetic relationship not smaller than 25%. There are many circumstances in which people call non-kin with a kin name. Malay people, for instance, refer to with whom they share food as kin (Shapiro 2011). On the Yap “a father” is a person with whom a child spends time and does activities (Schneider 1984). Cultural anthropologists call such cases fictive kinship, and sociologists use the term “chosen” or “voluntary” kin, and those terms refer to bonds including religious rituals, friendship, or reciprocal economic relationships (Ebaugh and Curry 2000). It is therefore a known phenomenon, but mainly discussed in the context of social ties or a level of cooperation between an individual or a family and fictive kin (Nakane 2021; Curry et al. 2012). What has been, however, neglected, and what we include in our hypothesis are the ecological or economic conditions in which such custom should be more prevalent, and would have stronger biological relevance. Furthermore there are no studies on the relative fitness consequences of this custom for an initiator (e.g., parents who want their common friend to be called an aunt for their child) and the recipient or a person that is bestowed with such a “privilege” to become a fictive kin. A potential recipient does not need to accept this “privilege,” particularly if the costs (material or emotional) can be too high. For instance when due to emotional burden a nurse does not want to be called a sister by a patient.

One crucial, evolutionary relevant aspect related to the prevalence of kin term mimicry in different societies might be socio-ecological conditions. Cooperation understood in the light of the Hamiltonian rule is known to be more beneficial in harsh environments (Andreas et al. 2007). Therefore, it is likely that in harsh, unpredictable environments, and in a high fertility society, kin term mimicry might be more frequent. Furthermore, we hypothesize that even within a society individuals with low/average income/wealth might be more likely to seek extra kin. It can be, for instance, confirmed by the fact that in many societies, especially among immigrants who live usually in poorer conditions, fictive kin (i.e., persons that are not genetically or marriage related) based on friendship or religious rituals are more frequent (Ebaugh and Curry 2000).

The hypotheses on the prevalence of kin naming mimicry might be related, however, not only to different ecological conditions e.g., harsh versus favorable environment, but also to an individual’s biological or psychological conditions. Still another issue to be addressed within the frame of KTM is related to symmetrical (as in the case of the relationship between adults) or asymmetrical (adults-children) potential costs that are born by fictive kin. In the latter case, it is obvious that the conscious initiators of attributing kin terms to non-kin are usually adults (e.g., parents or grandparents). Using KTM may help increase bonds, as fictive kin terminology helps an individual to affiliate with an appropriate group, and can help in potential cooperation (Ebaugh and Curry 2000; Dunbar 2008). The biological consequences in different combinations of KTM might of course differ. As in the case of all altruistic acts, in many societies being invited to be a fictive kin is supposed to be a privilege, and therefore hardly anyone refuses the “honor” of being named as another family member despite the cost this name might bring to the accepting person. To reject such an invitation can carry a social penalty. But accepting such an invitation does not necessarily require from a fictive kin to perform very well. There are studies suggesting that such gestures without actual efficient help can be enough (Burum et al. 2020). Those who choose a person to be a fictive kin for a child, for instance, usually think it over and consider only those, who they can rely on or expect some benefits for a child. Achieving benefits when attributing kin terms can be also characteristic in some social groups. For example, some religious groups choose mainly well educated and wealthy recruits, who when becoming members are later called with kin names (Qirko 2009). On a personal level, those who invite others to have a kin name (or a kin term that is related to a higher genetic coefficient), might also differ in personality and biological attributes from those who do not, or who do it only rarely.

Humans are aware of kinship ties, and they remember them well for their whole lives. What is, however, also very important is that humans typically attach many positive emotions (or respect) to such names, which we use for consanguineous kin, and some societies also foster very positive attitudes toward persons with kin terms, even after death (Bayliss 1973). In some societies such phenomenon can become a motivation to sacrifice one’s own life in case of revenge or protection of a kin or person with a kin term (Qirko 2009). This positive emotional attribution to close kin names seems to be a prerequisite for the emergence of KTM in our species.

Kin term mimicry is therefore not a deception (as any mimicry in other animals) or an attempt to cheat others, but usually a kind of conscious agreement that might be beneficial for both “sides” like it is in the Müllerian mimicry. Nevertheless, the benefits might not be equal, i.e., related to some resources (goods or time) exchanged at the same time, and might strongly depend on socio-ecological factors and age differences between fictive relatives. For instance, if a child calls its mother’s friend an aunt, the child might not even be aware of the kinship naming rules, and might have the same or even stronger affection for a fictive aunt than for their mother’s sister. This also means that such a child may rely on a fictive aunt’s help or generosity that will positively influence a child’s growth and development.

It is known that not only close genetic kinship is related to cooperation and helping (Kramer 2010). Such as altruism might be characterized as loss for one individual, reciprocal altruism occurs when two entities repeatedly exchange favors or benefits of equal fitness value (Qirko 2009). KTM in humans, however, may dramatically increase the proneness to help or cooperate, and therefore influence the intensity of social networks, the complexity of human interactions, and eventually human evolutionary success. KTM probably also enhances the frequency of reciprocal altruism (RA) in humans. Since kin names that are used to denote relation matter mainly by their emotional content (Boroditsky 2011), it increases the probability of reciprocity and decreases the risk of failing or avoiding reciprocal acts. It is known that religious institutions take over kinship terminology to their benefit (Rotkirch 2017). An institution or a country might be viewed as an entity (kin entity), with which a person might want to bond (mother land, fraternity and so on), and the strength of such bonding might depend on circumstances (Johnson 1987). The human tendency to bond and develop attachment can be used and manipulated by military, political or religious institutions to request resources, including a sacrifice of one’s own life. That might be seen as evolutionary maladaptive, but in the perspective of Hamilton’s rule, can be interpreted as serving all parties: recruit’s genetically related family (bringing monetary supply and/or respect), the organization (recruit’s resources), and recruit itself (hero status and/or afterlife reward) (Qirko 2009). Furthermore, people also react faster when solving moral dilemmas when it refers to a kin and not to a friend. The result is equal for genetically related kin and entities named as a kin (Machin and Dunbar 2015), which is attributed to how language influences the way we think and the way we behave. It can define what we remember, and how we organize memories (Boroditsky 2011). A certain word can give rise to a reaction of disgust or contentment (Gupta et al. 2007). We assume that a word chosen to describe a person of relation may determine the style of a relationship. A child may feel closer to a female adult when her “name” is “an aunt”, rather than when she is, for example, “Mrs Maria.” What is, however, interesting is that kinship terminology may evoke stronger positive emotional connotations in the first and last born than in the middleborns (Salmon 1998). Kinship terminology might be an emotional powerful tool. Kin terminology systems, may be based either on culture or biology (Cronk et al. 2019), and now kin related vocabulary is encoded in human psychology in a similar way to sex and age (Machin and Dunbar 2015). Cronk et al. (2019) argue that kin terminology must be an effect of biological processes as there are only six main systems. They point out that having such terminology may be useful if people want to make fitness enhancing behavior or manage conflicts. Such fitness interdependence can bring benefit to both entities or benefit to one side with a loss to another. This strongly suggests the adaptive meaning of KTM in enhancing social integrity.

Although reciprocal altruism and fictive kin phenomenon are well described in anthropological and evolutionary literature, it has been left unknown in which ecological, economical, psychological and sociological circumstances we should expect KTM to occur. We also do not know who usually initiates such denotation e.g., is it an adult or a child? And what is most important, there has not been enough scientific emphasis on biological benefits that the initiator and the recipient of KTM may gain. Taking under consideration the gap in understanding of this phenomenon, we would like to introduce the hypothesis stating that KTM is an evolutionary adaptive phenomenon whose prevalence could be studied in dependence of many testable factors, such as:

1. *Ecological/economic condition* In societies with low fertility and harsh, unpredictable environmental conditions, KTM will be more likely. Also, within society, people with lower income will be more prone to seek a fictive kin for their child.
2. *Individual biological quality* Lower biological quality of a person (or his/her children or in general descendants) will be related to proneness to impose kin terms on non-kin individuals.
3. *Number of children* More children is related to a higher cost for parents and the need for more resources, and therefore parents (or other family members) will be more prone to impose kin terms on some non-kin individuals.

We also propose a few additional hypotheses:

4. The preferable fictive kin will be the one with similar or slightly higher economic and social status. It might also be a person with lower family cost, and therefore with more surplus resources that can be dedicated to a fictive kin.
5. A fictive relative with a mimicked kin name provides financial or a psychological support to a fictive kin child, which is viewed/experienced as a significant support, and possibly increases a child’s fitness.
6. Being invited to be a fictive kin is seen as an honor and therefore is rarely rejected. This is a social rule that is also related to potential reciprocity.
7. In the fictive kin relationship between adult and child, it is a child that usually benefits, and it is an asymmetric relationship. In the case of adult-adult fictive kin, reciprocity is much more common and is a more symmetrical relationship. In the case of religious communities, the leaders (e.g., priests) usually benefit (in terms of resources or respect and social status) more than their fictive “sons” or “daughters.”
8. Imposed fictive kin terms may be also related to hierarchy in the group (or dominance–submission relationship), or to gaining a higher level of respect. In the former case when an older man calls a younger non-kin man “a son,” and in the latter case when a clergyman is called a “father” and therefore demands respect and may be treated as a more knowledgeable person.

We believe that all these hypotheses can be tested empirically in different societies or in cross-cultural studies. Due to different socio-ecological or cultural conditions, the results of such studies may of course differ in different societies. It is then obvious that not all hypotheses will be positively verified in each society.

## Data Availability

Not applicable.

## Footnotes

1 The term “Eskimo” has been used in the text as a label related to so called kin system. Choosing such vocabulary is based on established scholarly literature in the field. The term was not used in reference to people, as it is understood, that it could be perceived as pejorative.
